# Teaching an educational simulation elective outside the simulation center

**DOI:** 10.36834/cmej.70537

**Published:** 2020-09-23

**Authors:** A.J. Kleinheksel, Matthew Tews

**Affiliations:** 1 Medical College of Georgia, Augusta University, Georgia, USA

As medical education transitioned to online learning during the COVID-19 pandemic there was an immediate need for remote clinical education.^1^ One solution proposed at the Medical College of Georgia was to convert an existing elective on educational simulation. The elective was traditionally offered to no more than four students at a time in an interdisciplinary simulation center. Through the transition process two research questions arose: RQ1) Could the Simulation Education elective achieve the same learning objectives when removed from the experiential learning environment? And RQ2) Could the Simulation Education elective be successfully expanded to accommodate a larger number of students? The aim of this study was to determine both the efficacy of the first two online iterations of the course, but also to determine if the online elective could be offered successfully in future, if social distancing measures were once again required. If medical simulation content could be taught effectively in an online environment it would afford greater flexibility, both to accommodate public health safety and educate greater numbers of medical students on best practices in educational simulation to prepare them for their subsequent educator roles in residency and beyond. This study was approved by the Institutional Review Board of Augusta University.

As part of the month-long Simulation Education elective, each of the 26 students enrolled (8 in April and 18 in May) was provided with a Trello^TM^ board populated with 14 cards.^2^ Trello is a cloud-based project management software that allowed assignments (“cards”) to be moved to different states of completion: to do, in progress, ready for review, and approved. Both students and faculty could comment on each card both for general communication and with their assigned reflections. The intended learning outcomes of the traditional elective were to: 1) describe the theories supporting the use of simulation in education, 2) develop a simulation case using best practices, and 3) utilize technology to facilitate simulations. Although students could not run their completed case, they will be allowed to run them later, if desired. The traditional simulation observations were replaced with video recordings of control room equipment, a prebriefing, four simulation cases, and three debriefings. Each of the 14 Trello cards represented a learning objective for the elective, and contained several tasks to complete in their pursuit. An example is card #3, “describe the role of theory in educational simulation.” The card instructed the student to read three academic articles on the application of learning theory in simulation design, to find and attach an open education resource (OER) that contributed to their understanding, and to write a reflection and identify outstanding questions in a comment posted to the card. The culminating objective was to develop, present, and refine a new simulation case. Through the use of individual Trello boards, the elective could be delivered largely asynchronously with only three meetings: an introduction to the field of simulation, a discussion of ideas for case topics, and presentation of the completed simulation cases.

Both quantitative and qualitative methods will be employed to analyze the data currently being collected. Student evaluations of learning (SEL) will be analyzed using descriptive statistics. Content analysis^3^ will be conducted using the data collected from the comments, questions, and reflections written by students on the Trello boards, educator reflections, and each of the completed simulation cases. The qualitative content analysis will determine the extent to which learning objectives were achieved (RQ1), while both the SEL and qualitative themes will be used to evaluate the student perceptions of quality and efficacy of the elective (RQ2). Through descriptive statistics and content analysis, this study will determine if learning objectives were met, as well as identify any emergent themes relevant to the research questions.
